# Development of single-cell transcriptomic atlas of human plasmacytoid dendritic cells from people with HIV-1

**DOI:** 10.1016/j.xpro.2023.102777

**Published:** 2023-12-21

**Authors:** Lamin B. Cham, Lin Lin, Martin Tolstrup, Ole S. Søgaard

**Affiliations:** 1 Department of Infectious Diseases, Aarhus University Hospital, 8200 Aarhus, Denmark; 2 Department of Clinical Medicine, Aarhus University, 8200 Aarhus, Denmark; 3 Department of Biomedicine, Aarhus University, 8000 Aarhus, Denmark

**Keywords:** Bioinformatics, Immunology

## Abstract

Many immunological treatment strategies for reducing the HIV-1 reservoir and enhancing adaptive immunity aim at activating the human plasmacytoid dendritic cells (pDCs). Here, we present a protocol for pDC enrichment, single-cell analysis, and development of a pDC transcriptomic database from healthy individuals and people with HIV-1 before and after Toll-like receptor 9 agonist treatment.

For complete details on the use and execution of this protocol, please refer to Cham et al.^1^

## Highlights

• Human pDCs enrichment from healthy individuals and PWH before and after MGN1703 treatment
• scRNA library preparation, cluster analysis, and differential gene analysis
• Development of transcriptomic atlas of pDCs from healthy individuals vs. PWH
• Development of transcriptomic atlas of pDCs from PWH before and after MGN1703 treatment

## Abstract (editor highlights)

Publisher’s note: Undertaking any experimental protocol requires adherence to local institutional guidelines for laboratory safety and ethics.

## Before you begin

The protocol below describes human pDCs enrichment from peripheral blood mononuclear cells (PBMCs), single cell analysis, and development of human pDC transcriptomic database from healthy individuals and PWH.

Human pDCs constitute less than 0.5% of PBMCs^2^ and the percentage of circulating pDCs decreases during infection.^3^ In addition, pDCs are very fragile and a lot of cells are lost during sample processing.^4^ It is therefore essential to have adequate numbers of PBMCs to obtain enough pDCs for your single cells experiment. Performing dead cells removal prior to pDC enrichment is recommended to ensure that only live pDCs are enriched. Additionally, despite pDC enrichment there will be some contamination from other immune cells and cell death due to cellular stress. Therefore, it is important to perform appropriate quality control after sequencing and remove non-pDC clusters from your sc-RNA dataset. Development of pDC transcriptomic database should be done on sc-RNA dataset containing pDCs only.

### Institutional permission

Experiments on human samples, particularly PWH, must be performed in accordance with relevant institutional and national guidelines and regulations. Individual participant data cannot be made available due to Data Protection Regulations. A limited and completely anonymized version of human dataset can only be obtained upon request.

### Dead cells removal

**Timing: 1 h**

1. Thaw PBMCs and filter cells.
2. Removing dead cells using EasySep Dead Cell Removal kit.

### Human pDC enrichment

**Timing: 2 h**

3. pDC enrichment using human plasmacytoid dendritic cells isolation kit II.
4. Flow cytometry validation using pDC surface markers (CD303 and CD123).

### Single cell library preparation and sequencing

**Timing: 2–3 days**

5. Sc-RNA library construction using the Chromium Next GEM Single Cell 3**′** Reagent Kits v3.1.
  a. Master mix preparation and GEM loading.
  b. GEM generation and cleanup.
  c. cDNA amplification, cleanup, and quantification.
  d. Fragmentation, Post Fragmentation, End Repair & A-tailing Double Sided Size Selection.
  e. Adaptor Ligation and Post Ligation Cleanup.
  f. Sample Index PCR and Post Sample Index PCR Double Sided Size Selection.
  g. Post library construction and quality control.
6. Sequencing.

### Analysis of sc-RNA data

**Timing: 3–4 days**

7. Quality control of RNA-seq data.
8. Cluster analysis and selection.
9. pDC dataset cleanup.

### Development of pDC transcriptomic database

**Timing: 1–2 days**

10. Develop dataset of pDCs from healthy individual’s vs. PWH (https://dreamapp.biomed.au.dk/HIV_pDC/control_HIV_pDC/).
11. Develop dataset of pDCs from PWH before and after MGN1703 treatment (https://dreamapp.biomed.au.dk/HIV_pDC/HIV_TLR9_pDC).

## Key resources table

**Table:** 

REAGENT or RESOURCE	SOURCE	IDENTIFIER
**Antibodies**		

Chromium Next GEM Single Cell 3′ Kit v3.1	10× Genomics	PN-1000268
3′ Feature Barcode Kit	10× Genomics	PN-1000262
Chromium Next GEM Chip G Single Cell Kit	10× Genomics	PN-1000120
Dual Index Kit TT Set A	10× Genomics	PN-1000215
Dual Index Kit NN Set A	10× Genomics	PN-1000243
Dual Index Kit NT Set A	10× Genomics	PN-1000242
EasySep Dead Cell Removal (Annexin V) Kit	STEMCELL	Cat #:17899
Human plasmacytoid dendritic cells isolation kit II	Miltenyi Biotec	Cat #:130-092-207
Fixable near-IR (0.5 μL/sample)	Thermo Fisher Scientific	*Cat #: L10119*
Anti-Lin cocktail (3 μL/sample)	BioLegend	Cat #: 348801
Anti-CD303 (1 μL/sample)	Thermo Fisher Scientific	Cat #: 25-9818-42
Anti-CD123 (1 μL/sample)	Thermo Fisher Scientific	Cat #: 20-0116

**Deposited data**		

Raw and processed scRNA-seq data	This paper	GEO accession GSE228078
Codes for downstream analysis	This paper	https://github.com/laminbcham/sc-RNA-of-human-pDC-in-HIV-infection/blob/main/figure%20analysis%20codes

**Software and algorithms**		

R version 4.2.0		https://www.r-project.org/
Cell ranger count version 6.0.2	10× Genomics	https://support.10xgenomics.com/singlecell-geneexpression/software/downloads/3.0/
Seurat version 4.0.3	Seurat	https://cran.rproject.org/web/packages/ Seurat/index.html
DoubletFinder version 2.0.3	McGinnis et al.^5^	https://rdrr.io/github/chris-mcginnis-ucsf/DoubletFinder/
dplyr version 1.1.3	Mangiola et al.^6^	https://genomicsclass.github.io/book/pages/dplyr_tutorial.html
DESeq2 version 1.41.12	Liu et al.^7^	https://bioconductor.org/packages/release/bioc/html/DESeq2.html
ggplot2 version 3.4.4	Wu et al.^8^	https://altaf-ali.github.io/ggplot_tutorial/getting-started.html
BiocManager version 3. 15	Cattley et al.^9^	https://www.bioconductor.org/install/
limma version 3.46.0	Liu et al.^7^	https://bioconductor.org/packages/release/bioc/html/limma.html
ShinyCell version 2.1	Ouyang et al.^10^	https://github.com/SGDDNB/ShinyCell
FlowJo software version 10.8.2	FlowJo LLC, Ashland, OR, USA	FlowJo LLC

## Step-by-step method details

### Dead cells removal

**Timing: 1 h**

This section provides the requirements that are necessary to adequately remove dead cells from cryopreserved PBMCs.

1. Thaw PBMCs and filter cells.
  a. PBMCs can be thawed and suspend in to 1–2 mL buffer.
  b. Use 30 μm pre-separation filter to remove all debris.

***Note:*** Filtering cryopreserved PBMCs is highly recommended because of debris of dead cells or other substances as a result of preservation.

2. Removing dead cells using EasySep Dead Cell Removal kit.
  a. Cell pellets should be resuspended in 400 μL of 1× Binding Buffer.
  b. Add 100 μL of Dead Cell Removal Microbeads, mix and incubated for 15 min at room temperature (20°C−25°C).
  c. Suspend cells in 400 μL of 1× Binding Buffer and mix.
  d. Put tube in EasySep Magnet and incubate for 5 min.
  e. Pick up the magnet, and in one continuous motion invert the magnet and tube, pouring the enriched cell suspension into a new tube.

***Note:*** perform flow cytometry of PBMCs before and after dead cells removal to ensure that the dead cell removal is successful. This can be done by staining with viability dye (Near-IR) for 20 min at 4°C.

### Human pDC enrichment

**Timing: 2 h**

This section provides the requirements that are necessary to enrich human pDCs from PBMCs.

3. pDC enrichment using human plasmacytoid dendritic cells isolation kit II.
  a. Cells suspension obtained after dead cell removal should be kept in ice or 4°C.
  b. Centrifuge the cell suspension at 300 × *g* for 10 min and gently aspirate supernatant.
  c. Suspend cell pellet in 400 μL of buffer, add 100 μL of the Non-PDC Biotin-Antibody Cocktail I, mix and incubate for 10 min in ice or 4°C.
  d. Wash cells by adding at least 5 mL of buffer, centrifuge at 300 × *g* for 10 min and gently aspirate supernatant.
  e. Add 400 μL of buffer, add 100 μL of the Non-PDC MicroBead Cocktail II, mix and incubate for 15 min in ice or 4°C.
    ***Note:*** During incubation, please prepare MACS Column and MACS Separator. It is recommended to place LD Column in the MACS Separator and rinse with 2 mL of buffer.
  f. Again, wash cells by adding at least 5 mL of buffer, centrifuge at 300 × *g* for 10 min and gently aspirate supernatant.
  g. Suspend cells in 500 μL of buffer and proceed to magnetic separation.
  h. Put cell suspension in ice and or 4°C. Meanwhile, place LD Column in the MACS Separator and rinse with 2 mL of buffer (this step should be done during the last incubation).
  i. Put the cell suspension cell onto the column, collect unlabeled cells that pass through and wash column with 2 mL of buffer.
4. Flow cytometry validation using pDC surface markers (CD303 and CD123).
  ***Note:*** It is recommended but not required to perform flow cytometry of enriched cells to check the purity.
  a. Block non-specific binding and stain cells with pDC surface markers CD303 and CD123 antibodies for 30 min at 4°C.
  b. Perform flow cytometry and analyze pDC purity.
    ***Note:*** pDCs are fragile and should always be stored in ice or at 4°C. Start the single cell library preparation immediately after enrichment.

### Single cell library preparation and sequencing

**Timing: 2–3 days**

This section provides the requirements that are necessary to perform single RNA-seq of enriched pDCs. The volume of certain reagents in preparing master Mix depends on the number of reactions (e.g., 1×, 4× or 8×) described in detail in the 10× genomic manual.

5. Sc-RNA library construction using the Chromium Next GEM Single Cell 3**′** Reagent Kits v3.1.
  ***Note:*** Reagents and equipment’s should be prepared while enriching pDCs and start the single cell library preparation immediately after enrichment.
  a. Prepare reaction mix and GEM loading.
    i. Calculate the number of cells to be used and target cell recovery.
    ii. Prepare master mix on ice. The master should include RT Reagent B, Template Switch Oligo, Reducing Agent B and RT Enzyme B.
      **Table:** GEM master mix (for 1× reaction)

Reagent	Amount (μL)
RT Reagent B	18,8
Template Switch Oligo	2,4
Reducing Agent B	2,0
RT enzyme	8,7
Total	31,9

      ***Note:*** Pipette mix each reagent before adding to master mix. The volume for the number of reactions is well described in the 10× Genomic manual.
    iii. Add 50% glycerol solution to each unused well on the Next GEM Chip G. Add 70 μL in row 1, 50 μL in row 2 and 45 μL in row 3.
    iv. Prepare master mix with pDCs suspension and add appropriate volume of nuclease-free water and mix.
      ***Note:*** The volume of cell suspension and nuclease-free water depend on the cell concentration and well described in the 10× Genomic manual.
    v. Load 70 μL of pDCs + master mix in the row 1 of the Next GEM Chip G.
      ***Note:*** Vortex the Gel Beads and centrifuge shortly
    vi. Load 50 μL Gel Beads in row 2 of the Next GEM Chip G.
    vii. Load 45 μL Partitioning Oil in row 3 of the Next GEM Chip G.
    viii. Attached the GEM Gasket and place the Next GEM Chip G into the Chromium Controller and run.
  b. GEM generation and cleanup.
    i. Once the Chromium controller finished, remove the Chip G and place at 45 degree and gently aspirate 100 μL GEM from row 3 into new tubes.
      ***Note:*** Label the sample in each tube
    ii. Add 125 μL Recovery Agent to each sample at room temperature for 2 min or until there is separation of two layers of liquid.
    iii. Gently remove and discard 125 μL Recovery Agent/Partitioning Oil (pink) from the bottom of the tube.
      **CRITICAL:** DO NOT aspirate the aqueous solution, it contains your sample
    iv. Put sample in a RT-incubation preferably on Bio-Rad as shown below.
      **Table:** GEM-RT Incubation

Steps	Temperature	Time
1	53°C	45 min
2	85°C	5 min
3	4°C	Hold
    v. Prepare Dynabeads Cleanup Mix using Dynabead Myone silane, Reducing agent B and nuclease-free water. The volume depends on the number of reactions (e.g., 1×, 4× or 8×) described in detail in the 10× genomic manual.
      **Table:** Dynabeads Cleanup mix (for 1× reaction)

Reagent	Amount (μL)
Cleanup buffer	182
Dynabeads Myone silane	8
Reducing Agent B	5
Nuclease-free water	5
Total	200
    vi. Vortex and add 200 μL to each sample. Pipette mix and incubate at room temperature for 10 min.
    vii. Remove the supernatant, add 300 μL 80% ethanol to the pellet while on the magnet and discard ethanol. Repeat ethanol wash and air dry.
    viii. Prepare Elution Solution-I using buffer EB, 10% tween 20 and reducing agent B. The volume depends on the number of reactions (e.g., 1×, 4× or 8×) described in detail in the 10× genomic manual.
      **Table:** Elution Solution I (for 1× reaction)

Reagent	Amount (μL)
Buffer EB	98
10% Tween 20	1
Reducing agent	1
Total	100
    ix. Add 35.5 μL Elution Solution I, pipette mix and incubate for 2 min at room temperature.
    x. Transfer 35 μL sample to a new tube strip.
  c. cDNA amplification, cleanup, and quantification.
    i. Prepare cDNA Amplification reaction Mix using Amp mix and Feature cDNA primer 3. The volume depends on the number of reactions (e.g., 1×, 4× or 8×) described in detail in the 10× genomic manual.
      **Table:** cDNA Amplification Reaction Mix (for 1× reaction)

Reagent	Amount (μL)
Amp mix	50
Feature cDNA Primers 3	15
Total	100
    ii. Add 65 μL cDNA Amplification Reaction Mix to 35 μL sample, pipette mix and centrifuge briefly.
    iii. Incubate in a thermal cycler following protocol recommended in the 10× genomic manual.
      **Table:** cDNA Amplification thermal cycler (# of cycle is 11 or 12).

Steps	Temperature	Time
1	98°C	3 min
2	98°C	15 s
3	63°C	20 s
4	72°C	1 min
5	Next cycle	
6	72°C	1 min
7	4°C	Hold

      ***Note:*** You can stop and store sample at 4°C for up to 72 h or −20°C for ≤1 week or proceed to the next step.
    iv. Vortex to resuspend the SPRIselect reagent and add 60 μL SPRIselect reagent to each sample.
    v. Pipette mix and incubate for 5 min at room temperature. Then place on a magnet (high) until it’s clear. Remove supernatant from pellet.
      **CRITICAL:** DO NOT discard the pellet
    vi. Add 200 μL 80% ethanol to the pellet for 30 s, remove the ethanol and repeat wash.
    vii. Centrifuge briefly and place on magnet (low) for 2 min. Remove remaining ethanol and air dry.
    viii. Add 40.5 μL Buffer EB, mix and incubate for 2 min at room temperature.
    ix. Place on magnet (high) until the solution is clear.
    x. Transfer 40 μL sample to a new tube strip.
    xi. Run 1 μL of sample, diluted 1:10 on an Agilent Bioanalyzer High Sensitivity chip.
      **CRITICAL:** Please ensure that each sample yields a good cDNA concentration before proceeding.
  d. Post Fragmentation, End Repair & A-tailing Double Sided Size Selection.
    i. Use only 10 μL cDNA sample. This is enough to generate 3′ Gene expression library.
      ***Note:*** The remaining 30 μL cDNA sample should be stored in −20°C.
    ii. Prepare fragmentation mix using fragmentation buffer and fragmentation enzyme. The volume depends on the number of reactions (e.g., 1×, 4× or 8×) described in detail in the 10× genomic manual.
      **Table:** Fragmentation Mix (for 1× reaction)

Reagent	Amount (μL)
Fragmentation Buffer	5
Fragmentation Enzyme	10
Total	15
    iii. Add 25 μL Buffer EB to each sample, then add 15 μL Fragmentation mix, pipette mix and centrifuge briefly.
    iv. Transfer into thermal cycler following incubation protocol which is described in detail in the 10× genomic manual.
      **Table:** Fragmentation thermal cycler

Steps	Temperature	Time
Pre-cool block	4°C	Hold
Fragmentation	32°C	5 min
End repair and A-tailing	65°C	30 min
Hold	4°C	Hold
    v. Once the thermal cycler completes, vortex the SPRIselect and 30 μL to each sample and incubate for 5 min at room temperature.
    vi. Place samples in magnet (high) until the solution is clear and transfer 75 μL supernatant into new tubes.
    vii. Add 10 μL SPRIselect to the new tubes containing transferred supernatant and incubate for 5 min at room temperature.
    viii. Place samples in magnet (high) until the solution is clear and discard 80 μL supernatant.
      **CRITICAL:** Do not discard the bead, use tubes containing beads in the next step.
    ix. Add 125 μL ethanol to the pellet for 30 s, remove ethanol and repeat wash twice. Remove remaining ethanol and air dry.
    x. Add 50.5 μL Buffer EB to each sample, pipette mix and incubate for 2 min at room temperature.
    xi. Place samples in magnet (high) until the solution is clear and transfer 50 μL sample into new tubes.
  e. Adaptor Ligation and Post Ligation Cleanup.
    i. Prepare Adaptor Ligation mix using Ligation Buffer, DNA Ligase, and Adaptor Oligos. The volume depends on the number of reactions (e.g., 1×, 4× or 8×) described in detail in the 10× genomic manual.
      **Table:** Adaptor Ligation mix (for 1× reaction)

Reagent	Amount (μL)
Ligation buffer	20
DNA Ligase	10
Adaptor Oligos	20
Total	50
    ii. Add 50 μL Adaptor Ligation mix to the 50 μL sample and pipette mix.
    iii. Transfer to thermal cycles following incubation protocol which is described in detail in the 10× genomic manual.
      **Table:** Adaptor Ligation thermal cycler

Steps	Temperature	Time
1	20°C	15 min
2	4°C	Hold
    iv. Once the thermal cycle completes, add 80 μL SPRIselect reagent to each sample, pipette mix and incubate for 5 min at room temperature.
    v. Place on magnet (high) until the solution is clear, then remove the supernatant.
    vi. Add 200 μL ethanol to pellet for 30 s, remove ethanol and repeat wash twice. Remove remaining ethanol and air dry.
    vii. Add 30.5 μL Buffer EB and incubate for 2 min at room temperature.
    viii. Place on magnet (low) until the solution is clear and then transfer 30 μL supernatant to new tubes.
  f. Sample Index PCR and Post Sample Index PCR Double Sided Size Selection.
    i. Add 50 μL Amp mix to new tubes containing the transfer supernatant. Then add 20 μL of an individual Index TT Set A to each sample, pipette mix and centrifuge briefly.
      ***Note:*** Record the well ID of the Index TT Set A.
    ii. Incubate the sample in a thermal cycler following incubation protocol which is described in detail in the 10× genomic manual.
      **Table:** Sample Index PCR (# of cycle is 10–12)

Steps	Temperature	Time
1	98°C	45 s
2	98°C	20 s
3	63°C	30 s
4	72°C	20 s
5	Next cycle	
6	72°C	1 min
7	4°C	Hold
    iii. Once thermal cycler completes, add 60 μL SPRIselect reagent, pipette mix and incubate for 5 min at room temperature.
    iv. Place on magnet (high) until the solution is clear and transferred 150 μL supernatant to new tubes.
    v. Add 20 μL of SPRIselect to the new tubes containing the transferred supernatant, pipette mix and incubate for 5 min at room temperature.
    vi. Place on magnet (high) until the solution is clear and remove 165 μL supernatant and add 200 μL ethanol to the pellet for 30 s. Remove ethanol and repeat wash twice.
    vii. Place in magnet (low), remove excess ethanol and add 35.5 μL Buffer EB, pipette mix and incubate for 2 min at room temperature.
    viii. Place magnet (low) until the solution is clear, and transfer 35 μL to new tubes.
      ***Note:*** The transferred supernatant is used for quality control and sequencing. The remaining sample should be stored in −20°C.
  g. Post library construction quality control.
    i. Run 1 μL sample at 1:10 dilution on an Agilent Bioanalyzer High Sensitivity chip.
      ***Note:*** Determine the average fragment size from the Bioanalyzer trace. This will be used as the insert size for library quantification.
6. Sequencing.

***Note:*** Our samples were sent to the Department of Molecular Medicine at the Aarhus University Hospital for sequencing. Single-cell barcoded cDNA libraries were sequenced on an Illumina NovaSeq 6000 system (100-cycle cartridge) with a sequencing depth of at least 50,000 reads per cell. You can also send your samples for sequencing to other companies.

### Analysis of sc-RNA data

**Timing: 3–4 days**

In this section, we describe how to analyze sc-RNA-seq data. The protocol starts by sequence alignment of raw scRNA-seq fastq files from each sample generate the cell-genes count matrices. Importing filtered feature-barcode matrices, excluding bad-quality cells, non-expressed genes and doublets. Cluster analysis, annotation and visualization and selection of pDC clusters for in-depth characterization.

***Note:*** Step-by-step codes used to perform quality control, cluster analysis and pDC selection can be found in our GitHub repository: https://github.com/laminbcham/sc-RNA-of-human-pDC-in-HIV-infection/blob/main/figure%20analysis%20codes

DOI at Zenodo: https://zenodo.org/doi/10.5281/zenodo.10225477.

7. Alignment, Quality control of RNA-seq data.
  a. Individual raw scRNA-seq fastq files from each sample should be aligned against the human reference genome (GRCh38) through the cell ranger count pipeline (Cell Ranger version 3.1.0, 10× Genomics Technology), which will generate the cell-genes count matrices.
  b. Use R studio and the Seurat package (version 4.0.3) to perform a clean-up and quality control based on cellular expression of mitochondrial genes (cells with a percentage of mitochondrial genes >5% were discarded). Use DoubletFinder algorithm to remove doublets.

Script 1: Quality control and doublet finder and removal

#This script is adapted fromhttps://github.com/laminbcham/sc-RNA-of-human-pDC-in-HIV-infection/blob/main/figure%20analysis%20codes.

# Our study includes 8 samples (HC61, HC63, HC65, HC66, HIV112_V1, HIV113_V1, HIV109_V1, HIV103_V1). Quality control and doublet finder were performed in each sample.

# quality control for sample HIV103_V1

library(Seurat)

library(SeuratObject)

library(dplyr)

setwd("C:/Users/au672897/Desktop/pDC Sequence data")

HIV103_V1.data <- Read10X(data.dir = "Raw data-all/HIV103_V1/filtered_feature_bc_matrix")

HIV103_V1<-CreateSeuratObject(counts = HIV103_V1.data, project = "HIV103_V1", min.cells = 0, min.features = 200)

HIV103_V1[["percent.mt"]] <- PercentageFeatureSet(HIV103_V1, pattern = "ˆMT-")

HIV103_V1 <- subset(HIV103_V1, subset = nFeature_RNA > 200 & nFeature_RNA < 2500 & percent.mt <5)

HIV103_V1 <- NormalizeData(HIV103_V1, normalization.method = "LogNormalize", scale.factor = 10000)

# Doublet finder and removal for sample HIV103_V1

remotes::install_github('chris-mcginnis-ucsf/DoubletFinder')

suppressMessages(require(DoubletFinder))

sweep.res.list_HIV103_V1 <- paramSweep_v3(HIV103_V1, PCs = 1:10, sct = TRUE)

sweep.stats_HIV103_V1 <- summarizeSweep(sweep.res.list_HIV103_V1, GT = FALSE)

bcmvn_HIV103_V1 <- find.pK(sweep.stats_HIV103_V1)

annotations <- HIV103_V1@meta.data$RNA_snn_res.0.5

homotypic.prop <- modelHomotypic(annotations)

nExp_poi <- round(0.075∗nrow(HIV103_V1@meta.data))

nExp_poi.adj <- round(nExp_poi∗(1-homotypic.prop))

HIV103_V1 <- doubletFinder_v3(HIV103_V1, PCs = 1:10, pN = 0.25, pK = 0.09, nExp = nExp_poi, reuse.pANN = FALSE, sct = TRUE)

head(HIV103_V1[[]])

# this function will provide the pANN and DF. Classification number

HIV103_V1 <- doubletFinder_v3(HIV103_V1, PCs = 1:10, pN = 0.25, pK = 0.09, nExp = nExp_poi.adj, reuse.pANN = "pANN_0.25_0.09_794", sct = TRUE)

Idents(HIV103_V1) <- "DF.classifications_0.25_0.09_794"

DimPlot(HIV103_V1, reduction = "umap")

HIV103_V1

DF.name = colnames(HIV103_V1@meta.data)[grepl("DF.classifications_0.25_0.09_794", colnames(HIV103_V1@meta.data))]

HIV103_V1_singlet = HIV103_V1[,HIV103_V1@meta.data[,DF.name] == "Singlet"]

HIV103_V1_singlet

saveRDS(HIV103_V1_singlet, file = "C:/Users/au672897/Desktop/pDC Sequence data/Singlet/HIV103_V1.rds")

8. Unsupervised Cluster analysis.
  a. Use the ‘FindIntegrationAnchors’ Seurat function for integration of samples.
  b. Use applied graph-based clustering and a non-linear dimension reduction using uniform manifold approximation and projection (UMAP) for cell cluster visualization.
  c. Perform differentially expressed genes to identify classical genes for each cluster.
  d. Use “AverageExpression” function, the ‘FindAllMarkers’ Seurat function for the annotation of cluster.

Script 2: Sc-RNA data integration and cluster analysis

#This script is adapted fromhttps://github.com/laminbcham/sc-RNA-of-human-pDC-in-HIV-infection/blob/main/figure%20analysis%20codes.

# sc-RNA data integration

library(Seurat)

library(SeuratObject)

library(dplyr)

library(pheatmap)

library(BiocManager)

library(ggplot2)

anchors <- FindIntegrationAnchors(object.list = list(HC61, HC63, HC65, HC66, HIV112_V1, HIV113_V1, HIV109_V1, HIV103_V1), dims = 1:20)

control_HIV <- IntegrateData(anchorset = anchors, dims = 1:20)

DefaultAssay(object = control_HIV) <- "integrated"

saveRDS(control_HIV, file = "C:/Users/au672897/Desktop/pDC Sequence data/pDC only/control_HIV.rds")

control_HIV <- readRDS("C:/Users/au672897/Desktop/pDC Sequence data/pDC only/control_HIV.rds")

#cluster analysis

control_HIV <- NormalizeData(control_HIV, normalization.method = "LogNormalize", scale.factor = 10000)

control_HIV <- ScaleData(object = control_HIV, verbose = FALSE)

control_HIV <- RunPCA(object = control_HIV, npcs = 30, verbose = FALSE)

control_HIV <- RunUMAP(object = control_HIV, reduction = "pca", dims = 1:20)

control_HIV <- FindNeighbors(object = control_HIV, reduction = "pca", dims = 1:20)

control_HIV <- FindClusters(control_HIV, resolution = 0.5)

DimPlot(control_HIV, reduction = "umap", label = TRUE, repel = TRUE, raster=FALSE, label.size = 10, pt.size = 0.5)

VlnPlot(control_HIV, features = c("CLEC4C", "IL3RA"), pt.size = 0)

FeaturePlot(control_HIV, features = c("CLEC4C", "IL3RA", "LILRA4"), raster=FALSE)

new.cluster.ids <- c("pDC","pDC","pDC", "B cells", "pDC","pDC", "NK/NKT cells","Erythroid-like cells", "T cells","Monocytes","T cells","Platelet","B cells","pDC","Platelet", "NK/NKT cells", "NK/NKT cells")

names(new.cluster.ids) <- levels(control_HIV)

control_HIV <- RenameIdents(control_HIV, new.cluster.ids)

9. pDC dataset cleanup.
  ***Note:*** Since pDC does not yield 100% purity and as shown in that there is contamination from other immune cells in the dataset, it’s important to select the pDC cluster for further in-depth analysis.
  a. Once cluster annotation is complete, use ‘’Subset’’ function to select the pDC cluster and set DefaultAssay as RNA.
    Script 3: Selection of pDC cluster
    #This script is adapted fromhttps://github.com/laminbcham/sc-RNA-of-human-pDC-in-HIV-infection/blob/main/figure%20analysis%20codes.
    # subset of pDC cluster
    control_HIV$pDC <- "other"
    control_HIV$pDC[which(control_HIV$integrated_snn_res.0.3 %in% c(0,1,2))] <- "pDC"
    Idents(object = control_HIV) <- "pDC"
    Idents(object = control_HIV, cells = 1:10) <- "pDC"
    control_HIV_pDC <- subset(control_HIV, idents = "pDC")
    control_HIV
    control_HIV_pDC
    saveRDS(control_HIV_pDC, file = "C:/Users/au672897/Desktop/pDC Sequence data/pDC only/control_HIV_pDC.rds")
  b. Perform re-cluster of the pDCs and analyze each pDC cluster base on the known pDC classical genes.
  c. Use “AverageExpression” function, the ‘FindAllMarkers’ Seurat function for the annotation of pDC clusters.

Script 4: Re-clustering of pDCs and DEG analysis of pDC

#This script is adapted fromhttps://github.com/laminbcham/sc-RNA-of-human-pDC-in-HIV-infection/blob/main/figure%20analysis%20codes.

# pDC clusters and DEG analysis

control_HIV_pDC <- readRDS("C:/Users/au672897/Desktop/pDC Sequence data/pDC only/control_HIV_pDC.rds")

control_HIV_pDC <- FindVariableFeatures(object = control_HIV_pDC, selection.method = "vst", nfeatures = 1000)

control_HIV_pDC <- ScaleData(object = control_HIV_pDC, features = rownames(control_HIV_pDC), verbose = FALSE)

control_HIV_pDC <- RunPCA(object = control_HIV_pDC, npcs = 30, verbose = FALSE)

control_HIV_pDC <- RunUMAP(object = control_HIV_pDC, reduction = "pca", dims = 1:20)

control_HIV_pDC <- FindNeighbors(object = control_HIV_pDC, reduction = "pca", dims = 1:20)

control_HIV_pDC <- FindClusters(control_HIV_pDC, resolution = 0.2)

control_HIV_pDC<-RenameIdents(control_HIV_pDC, "0"= "pDC1", "1"= "pDC2", "2"= "Exhausted pDC")

### Development of pDC transcriptomic database

**Timing: 1–2 days**

Here, we used **ShinyCell**, an R package that allows us to create interactive Shiny-based web applications to visualize single-cell data. This allows us to visualize and compare cell information and gene expression on reduced dimensions (e.g., UMAP), visualize the distribution of continuous cell information (e.g., nUMI, gene expression level) using violin plots or boxplots and visualize the expression of multiple genes using bubble plots or heatmap.^11^

We first developed database of pDCs from healthy individuals compared to PWH (https://dreamapp.biomed.au.dk/HIV_pDC/control_HIV_pDC/). Next, we developed another database of pDCs from PWH before and after MGN1703 treatment (https://dreamapp.biomed.au.dk/HIV_pDC/HIV_TLR9_pDC/). Both databases were generated and modified with ShinyCell, and then deployed to pre-established shiny server in Aarhus University.

***Note:*** We describe the Step-by-step codes used in the development of pDC transcriptomic database. This includes loading data and creating ShinyCell configuration, modifying the metadata and color palette, changing order of appearance of metadata, generation of Shiny app and different visualization in the Shiny app. Detail can be found in our GitHub repository: https://github.com/laminbcham/pDC-transcriptomic-database/edit/main/README.md

10. Development of transcriptomic atlas of pDCs from healthy individuals vs. PWH.

Script 5: Control vs. HIV pDC database

#This script is adapted fromhttps://github.com/laminbcham/pDC-transcriptomic-database/blame/main/README.md.

#control_HIV_pDC

library(Seurat)

library(ShinyCell)

setwd("D:/New folder/Analysis-HIV_lamin/10, DB")

seu <- readRDS("../RDS files from Lamin/control_HIV_pDC.rds")

head(seu[[]])

seu

DefaultAssay(seu) <- "RNA"

scConf_sp = createConfig(seu,maxLevels = 200)

scConf_sp = scConf_sp[c(1:4,10,11,35,36),]

makeShinyCodes(shiny.title = "control_HIV_pDC_Database",shiny.footnotes = "",defPtSiz = 0.1,

 shiny.prefix = "sp1", shiny.dir = "control_HIV_pDC/")

makeShinyFiles(seu,scConf_sp,gex.assay = "RNA",

 gex.slot = "data",

 gene.mapping = FALSE,

 shiny.prefix = "sp1",

 shiny.dir = "control_HIV_pDC/",

 default.multigene = NA,

 default.dimred = NA,

 chunkSize = 500)

11. Development of transcriptomic atlas of pDCs from PWH before and after MGN1703 treatment.

Script 6: HIV before vs. after TLR9 (MGN1703) pDC database

#This script is adapted fromhttps://github.com/laminbcham/pDC-transcriptomic-database/blame/main/README.md.

#HIV_TLR9_pDC

library(Seurat)

library(ShinyCell)

setwd("D:/New folder/Analysis-HIV_lamin/10, DB")

seu <- readRDS("../RDS files from Lamin/HIV_TLR9_pDC.rds")

head(seu[[]])

seu

DefaultAssay(seu) <- "RNA"

scConf_sp = createConfig(seu,maxLevels = 200)

scConf_sp = scConf_sp[c(1:4,10,11,35,36),]

makeShinyCodes(shiny.title = "HIV_TLR9_pDC_Database",shiny.footnotes = "",defPtSiz = 0.1,

 shiny.prefix = "sp1", shiny.dir = "HIV_TLR9_pDC/")

makeShinyFiles(seu,scConf_sp,gex.assay = "RNA",

 gex.slot = "data",

 gene.mapping = FALSE,

 shiny.prefix = "sp1",

 shiny.dir = "HIV_TLR9_pDC/",

 default.multigene = NA,

 default.dimred = NA,

 chunkSize = 500)

## Expected outcomes

Our protocol describes methods of human pDC enrichment, single cells RNA-seq analysis and development of human pDC transcriptomic database from healthy, PWH before and after TLR9 agonist (MGN1703) treatment. Following the step-by-step above, you can expect to successfully enrich enough live pDCs for your single cells experiment and how to construct a database for your single cell transcriptomic data. Development of pDC transcriptomic atlas helps easy understanding for non-computational researchers.

## Quantification and statistical analysis

Differential expression genes (DEGs) were analyzed using three tests, Wilcoxon-ranked sum test, t-test and t-test overestimated variance. DEGs were computed using the ‘FindMarker’ function of Seurat and the probability values were estimated with respect to all other clusters within each dataset.

## Limitations

Human pDCs only make up 0.1%–0.5% of all PBMCs and large blood volumes are required to obtain enough cells for the experiments. Enrichment of pDCs contains a lot of infiltrating immune cells. Time of sample collection is important to target interferon genes. Using cryopreserved PBMCs for pDC experiment is not very ideal, rather freshly isolated PBMCs is preferable.

## Troubleshooting

### Problem 1

Cryopreserved PBMCs yield poor cell viability.

### Potential solution

Use freshly isolated PBMC.

### Problem 2

Lot of infiltrating immune cells in the enriched pDC.

### Potential solution

Perform cell sorting instead of cell enrichment.

### Problem 3

Impossible or hard to perform surface protein analysis along with gene expression on pDCs.

### Potential solution

Ensure that enough PBMCs are available for pDC isolation.

### Problem 4

Quality control of raw pDC sequence data has high mitochondria markers.

### Potential solution

Perform single cell RNA library preparation immediately after isolation.

### Problem 5

Contamination of other immune clusters.

### Potential solution

Perform cluster selection and cleanup.

## Resource availability

### Lead contact

Individual participant data cannot be made available due to EU Data Protection Regulations (GDPR). A limited and completely anonymized version of the dataset can be obtained upon request. Further information and requests for resources and reagents should be directed to and will be fulfilled by the lead contact (Ole S. Søgaard): olesoega@rm.dk.

### Materials availability

pDC transcriptomic database of Healthy individuals vs. PWH on ART treatment https://dreamapp.biomed.au.dk/HIV_pDC/control_HIV_pDC/.

pDC transcriptomic database of PWH before vs. after MGN1703 treatment https://dreamapp.biomed.au.dk/HIV_pDC/HIV_TLR9_pDC/.

### Data and code availability

Single cell RNA-seq raw and processed data have been deposited at Gene Expression Omnibus (GEO accession GSE228078) https://www.ncbi.nlm.nih.gov/geo/query/acc.cgi?acc=GSE228078.

Original codes used to generate data of this paper are publicly available at GitHub: https://github.com/laminbcham/sc-RNA-of-human-pDC-in-HIV-infection/blob/main/figure%20analysis%20codes and the version of record is archived at Zenodo. DOI at Zenodo: https://zenodo.org/doi/10.5281/zenodo.10225477.

Any additional information required to reanalyze the data reported in this paper is available from the lead contact upon request.

## References

[1] Cham L.B., Gunst J.D., Schleimann M.H., Frattari G.S., Rosas-Umbert M., Vibholm L.K., van der Sluis R.M., Jakobsen M.R., Olesen R., Lin L. (2023). Single Cell Analysis Reveals a Subset of Cytotoxic-like Plasmacytoid Dendritic Cells in People with HIV-1. iScience.

[2] Li G., Cheng L., Su L. (2021). Phenotypic and Functional Study of Human Plasmacytoid Dendritic Cells. Curr. Protoc..

[3] Cham L.B., Pahus M.H., Grønhøj K., Olesen R., Ngo H., Monrad I., Kjolby M., Tolstrup M., Gunst J.D., Søgaard O.S. (2021). Effect of Age on Innate and Adaptive Immunity in Hospitalized COVID-19 Patients. J. Clin. Med..

[4] Gerrits J.H., Athanassopoulos P., Vaessen L.M.B., Klepper M., Weimar W., van Besouw N.M. (2007). Peripheral blood manipulation significantly affects the result of dendritic cell monitoring. Transpl. Immunol..

[5] McGinnis C.S., Murrow L.M., Gartner Z.J. (2019). DoubletFinder: Doublet Detection in Single-Cell RNA Sequencing Data Using Artificial Nearest Neighbors. Cell Syst..

[6] Mangiola S., Doyle M.A., Papenfuss A.T. (2021). Interfacing Seurat with the R tidy universe. Bioinformatics.

[7] Liu S., Wang Z., Zhu R., Wang F., Cheng Y., Liu Y. (2021). Three Differential Expression Analysis Methods for RNA Sequencing: limma, EdgeR, DESeq2. J. Vis. Exp..

[8] Wu X., Sui Z., Zhang H., Wang Y., Yu Z. (2020). Integrated Analysis of lncRNA-Mediated ceRNA Network in Lung Adenocarcinoma. Front. Oncol..

[9] Cattley S., Arthur J.W. (2007). BioManager: the use of a bioinformatics web application as a teaching tool in undergraduate bioinformatics training. Brief. Bioinform..

[10] Ouyang J.F., Kamaraj U.S., Cao E.Y., Rackham O.J.L. (2021). ShinyCell: simple and sharable visualization of single-cell gene expression data. Bioinformatics.

[11] Patil A.R., Kumar G., Zhou H., Warren L. (2023). scViewer: An Interactive Single-Cell Gene Expression Visualization Tool. Cells.

